# Potential Roles of Large Language Models in the Production of Systematic Reviews and Meta-Analyses

**DOI:** 10.2196/56780

**Published:** 2024-06-25

**Authors:** Xufei Luo, Fengxian Chen, Di Zhu, Ling Wang, Zijun Wang, Hui Liu, Meng Lyu, Ye Wang, Qi Wang, Yaolong Chen

**Affiliations:** 1 Evidence-Based Medicine Center School of Basic Medical Sciences Lanzhou University Lanzhou China; 2 World Health Organization Collaboration Center for Guideline Implementation and Knowledge Translation Lanzhou China; 3 Institute of Health Data Science Lanzhou University Lanzhou China; 4 Key Laboratory of Evidence Based Medicine and Knowledge Translation of Gansu Province Lanzhou University Lanzhou China; 5 Research Unit of Evidence-Based Evaluation and Guidelines, Chinese Academy of Medical Sciences (2021RU017) School of Basic Medical Sciences Lanzhou University Lanzhou China; 6 School of Information Science & Engineering Lanzhou University Lanzhou China; 7 School of Public Health Lanzhou University Lanzhou China; 8 Department of Health Research Methods, Evidence and Impact Faculty of Health Sciences McMaster University Hamilton, ON Canada; 9 McMaster Health Forum McMaster University Hamilton, ON Canada

**Keywords:** large language model, ChatGPT, systematic review, chatbot, meta-analysis

## Abstract

Large language models (LLMs) such as ChatGPT have become widely applied in the field of medical research. In the process of conducting systematic reviews, similar tools can be used to expedite various steps, including defining clinical questions, performing the literature search, document screening, information extraction, and language refinement, thereby conserving resources and enhancing efficiency. However, when using LLMs, attention should be paid to transparent reporting, distinguishing between genuine and false content, and avoiding academic misconduct. In this viewpoint, we highlight the potential roles of LLMs in the creation of systematic reviews and meta-analyses, elucidating their advantages, limitations, and future research directions, aiming to provide insights and guidance for authors planning systematic reviews and meta-analyses.

## Introduction

A systematic review is the result of a systematic and rigorous evaluation of evidence, which may or may not include a meta-analysis [1]. Owing to the strict methodology and comprehensive summary of evidence, high-quality systematic reviews are considered the highest level of evidence, positioned at the top of the evidence pyramid [2]. Additionally, high-quality systematic reviews and meta-analyses are often used to support the development of clinical practice guidelines, aid clinical decision-making, and inform health care policy formulation [3]. Currently, the methods of systematic reviews and meta-analyses are applied in various disciplines in medicine and beyond such as law [4], management [5], and economics [6], and have yielded positive results, contributing to the continuous advancement of these fields [7].

The process of conducting systematic reviews demands a substantial investment in terms of time, resources, human effort, and financial capital [8]. To expedite the development of systematic reviews and meta-analyses, various automated or semiautomated tools such as Covidence have been developed [9,10]. However, the emergence of large language models (LLMs), particularly chatbots such as GPT, presents a set of both challenges and opportunities in the realm of systematic reviews and meta-analyses [11]. Based on the emerging literature in this field, we here provide our perspectives on the potential for harnessing the capabilities of LLMs to accelerate the production of systematic reviews and meta-analyses, while also scrutinizing the potential impacts and delineating the crucial steps involved in this process.

## The Process and Challenges of Performing a Systematic Review and Meta-Analysis

**Figure 1:**
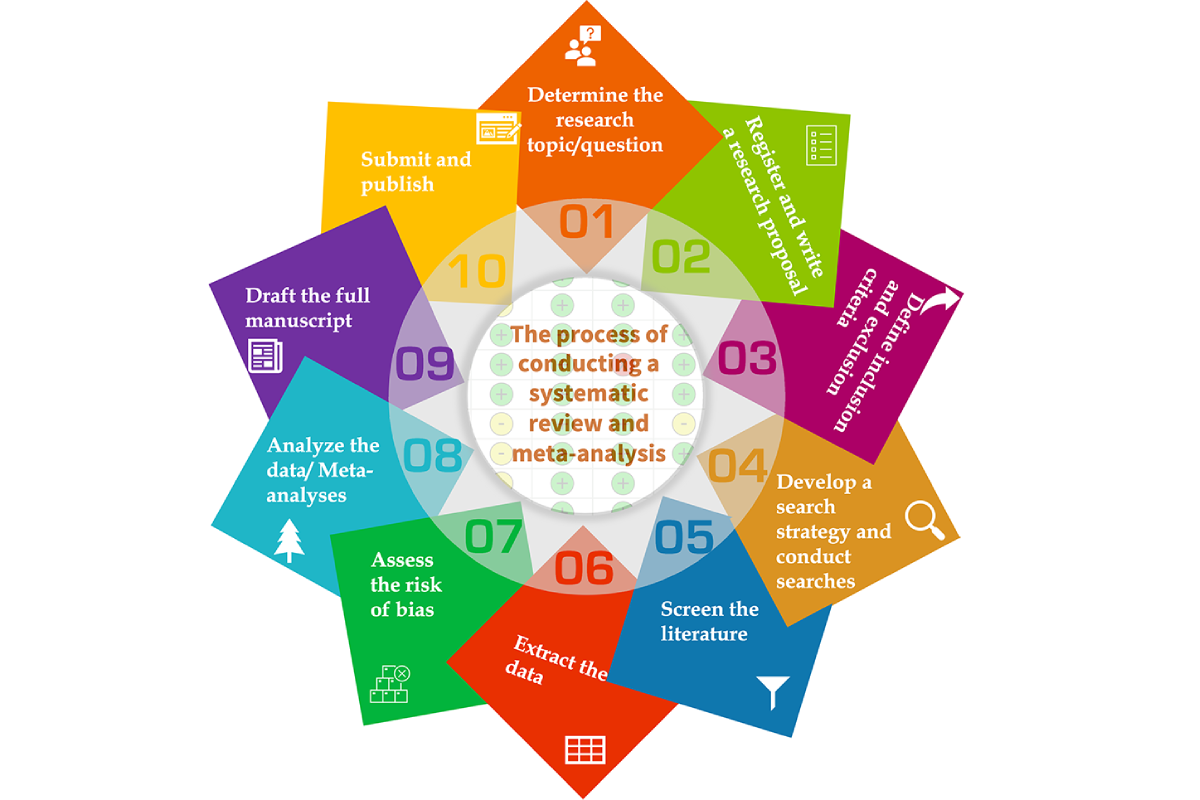
The process of conducting a systematic review and meta-analysis.

The procedures and workflows for conducting systematic reviews and meta-analyses are well-established. Currently, researchers often refer to the Cochrane Handbooks recommended by the Cochrane Library for intervention or diagnostic reviews [12,13]. In addition, some scholars and institutions have developed detailed guidelines on the steps and methodology for performing systematic reviews and meta-analyses [14-17]. Generally speaking, researchers should take the following steps to produce a high-quality systematic review and meta-analysis: determine the clinical question, register and draft a protocol, set inclusion and exclusion criteria, develop and implement a search strategy, screen the literature, extract data from included studies, assess the quality and risk of bias of included studies, analyze and processed data, write up the full text of the manuscript, and submit the manuscript for publication, as illustrated in Figure 1. These different steps contain many subtasks; therefore, conducting a complete systematic review and meta-analysis requires fairly complex and time-consuming work.

Although systematic reviews and meta-analyses have been widely applied and play an important role in developing guidelines and informing clinical decision-making, their production process faces many challenges. One of these challenges is the long production time and large resource requirements. The average estimated time to complete and publish a systematic review is 67.3 weeks, requiring 5 researchers and costing approximately US $140,000 [18,19]. More recently, the development of automated and semiautomated tools using natural language processing and machine learning have accelerated systematic review and meta-analysis production to some extent [20], with studies showing that such tools can help to produce a systematic review and meta-analysis within 2 weeks [21]. However, these tools also have some limitations. First, no single tool can fully accelerate the entire production process of systematic reviews and meta-analyses. Second, these tools cannot process and analyze literature written in different languages. Finally, the reliability of the results generated by these automated and semiautomated tools needs further validation as they are not yet widely adopted for this purpose.

## Applications of LLMs in Medical Research

Chatbots based on LLMs such as ChatGPT, Google Gemini, and Claude have become widely applied in medical research. These chatbots have proven to be valuable in tasks ranging from knowledge retrieval, language refinement, content generation, and medical exam preparation to literature assessment. ChatGPT has been shown to excel in accuracy, completeness, nuance, and speed when generating responses to clinical inquiries in psychiatry [22]. Moreover, LLMs such as ChatGPT play a pivotal role in automating the evaluation of medical literature, facilitating the identification of accurately reported research findings [23]. Despite their significant contributions, these chatbots are not without limitations. Challenges such as the potential for generating misleading content and susceptibility to academic deception necessitate further scholarly discourse on effective mitigation strategies. Standardized reporting practices may contribute to delineating the applications of ChatGPT and mitigating research biases [24].

ChatGPT has also demonstrated significant application potential and promise in the process of conducting systematic reviews and meta-analyses. Various studies [11,25-32] indicate that ChatGPT can play a pivotal role in formulating clinical questions, determining inclusion and exclusion criteria, screening literature, assessing publications, generating meta-analysis code, and assisting the full-text composition, among other relevant tasks. The details of these capabilities are summarized in Table 1.

**Table 1:** The possible functions of chatbots in the creation of systematic reviews and meta-analyses encompassing separate stages of the process.

Tasks	Potential roles and application steps of chatbots	References
Determine the research topic/question	Identify previously published systematic reviews and meta-analyses on the same topic. Assist in determining the rationale for the research question. Clarify the PICO (Population, Intervention, Comparison, Outcome) question.	[11,33-35]
Register and write a research proposal	Generate preliminary, unverified registration information. Draft an initial research proposal, subject to validation.	[11,36,37]
Define inclusion an exclusion criteria	Establish inclusion criteria. Establish exclusion criteria.	[11,38]
Develop a search strategy and conduct searches	Develop and optimize search strategies. Implement retrieval. Collect grey literature.	[11,25,29,33,39-42]
Screen the literature	Remove duplicate records. Screen literature titles, abstracts, and keywords. Screen the full text of the obtained literature. Download the full text of the literature.	[11,25,27,28,33,34,43-47]
Extract the data	Extract basic information. Extract patient information. Extract outcome information. Extract table information.	[11,25,26,47-50]
Assess the risk of bias	Extract relevant information based on the scale. Evaluate the risk of bias based on the scale. Present visual results.	[26,51-53]
Analyze the data/meta-analyses	Extract outcome information. Generate figures and tables for some results.	[11,26,37,54]
Draft the full manuscript	Search for relevant references. Polish language and grammar. Adjust the reference citation format. Summarize the abstract.	[25,33,55-58]
Submit and publish	Assist in selecting a suitable journal. Adjust the manuscript format. Compose a cover letter. Assist in preparing the submission.	[33,59]

## Potential Roles of LLMs in Producing Systematic Reviews and Meta-Analyses

### Determine the Research Topic/Question

Determining the clinical question of interest represents the initial and paramount step in the process of conducting systematic reviews and meta-analyses. At this juncture, it is crucial to ascertain whether comparable systematic reviews and meta-analyses have already been published and to delineate the scope of the forthcoming review and meta-analysis. Generally, for interventional systematic reviews, the Patient, Intervention, Comparison, and Outcome (PICO) framework is considered for defining the scope and objectives of the research question [60]. In this context, ChatGPT serves a dual role. On the one hand, it expeditiously aids in searching for published systematic reviews and meta-analyses related to the relevant topics (see Multimedia Appendix 1 and Multimedia Appendix 2) [34]. On the other hand, ChatGPT assists in refining the clinical question that needs to be addressed (see Multimedia Appendix 3), facilitating prompt determination of the feasibility of undertaking the proposed study. However, it is important to be cautious of the retrieval of false literature [35].

### Register and Write a Research Proposal

The registration and proposal writing process constitutes a pivotal preparatory phase for conducting systematic reviews and meta-analyses. Registration enhances research transparency, fosters collaboration among investigators, and mitigates the redundancy of research endeavors. Drafting a proposal helps in elucidating the research objectives and methods, providing robust support for the smooth execution of the study. For LLMs, generating preliminary registration information and initial proposal content is remarkably convenient and facile (see Multimedia Appendix 4 and Multimedia Appendix 5). For example, ChatGPT can assist researchers in generating the statistical methods for a research proposal [37]. However, considering that LLMs often generate fictitious literature, the content they produce may be inaccurate; thus, discernment and validation of the generated content remain essential considerations.

### Define Inclusion and Exclusion Criteria

The inclusion and exclusion criteria for systematic reviews and meta-analyses are instrumental in determining the screening standards for studies. Therefore, strict and detailed inclusion and exclusion criteria contribute to the smooth and high-quality conduct of preparing systematic reviews and meta-analyses. The use of a chatbot based on LLMs can help in establishing the inclusion and exclusion criteria (see Multimedia Appendix 6) [38]; however, the inclusion criteria need to be optimized and adjusted according to the specific research objectives and the exclusion criteria should be based on the foundation of the inclusion criteria. Therefore, manual adjustments and optimizations are also necessary.

### Develop a Search Strategy and Conduct Searches

ChatGPT can assist in formulating search strategies, using PubMed as an example [40]. Researchers can simply list their questions using the PICO framework and a search strategy can be quickly generated (Multimedia Appendix 1 and Multimedia Appendix 2). Based on the generated search strategy, one method is to copy the strategy from ChatGPT and paste it into the PubMed search box for direct retrieval [40,41]. Another approach involves using the OpenAI application programming interfaces (APIs) to invoke PubMed APIs with the search strategy generated by ChatGPT. This facilitates searching the PubMed database, obtaining search results, and applying predetermined inclusion and exclusion criteria. Subsequently, ChatGPT can be used to filter the search results, exporting and recording the filtered results in JSON format. This integrated process encompasses search strategy formulation, retrieval, and filtering. However, the direct use of LLMs to generate search strategies and complete the one-stop process of searching and screening may not yet be mature, and this poses a significant challenge for generating the PRISMA (Preferred Reporting Items for Systematic reviews and Meta-Analyses) flowchart. Therefore, we suggest using LLMs to generate search strategies, which should then be optimized and modified by librarians and computer experts (specializing in LLMs) before manually searching the databases. Additionally, to use search strategies transparently and reproducibly, the detailed prompts used should be reported [40,42].

### Screen the Literature

Literature screening is one of the most time-consuming steps in the creation of systematic reviews and meta-analyses. Prior to the advent of ChatGPT, there were already many automated and semiautomated tools available for literature screening, such as Covidence, EPPI-Reviewer, DistillerSR, and others [39]. With the emergence of ChatGPT, researchers can now train the model based on predefined inclusion criteria. Subsequently, ChatGPT can be used to automatically screen records retrieved from databases and obtain the filtered results. Previous studies suggested that using ChatGPT in the literature selection process for a meta-analysis substantially diminishes the workload while preserving a recall rate on par with that of manual curation [28,44-47].

### Extract the Data

Data extraction involves obtaining information from primary studies and serves as a primary source for systematic reviews and meta-analyses. Generally, when conducting systematic reviews and meta-analyses, basic information must be extracted from the original studies, such as publication date, country of conduct, and the journal of publication. Additionally, characteristics of the population, such as patient samples, age, gender/sex, and outcome data, are also extracted, including event occurrences, mean change values, and total sample size. Currently, tools based on natural language processing and LLMs, such as ChatGPT and Claude, demonstrate high accuracy in extracting information from PDF documents (see Multimedia Appendix 7 for an example) [47-50]. However, it is important to note that despite their promising capabilities, manual verification remains a necessary step in the data extraction process when using these artificial intelligence (AI) tools [61]. Using LLMs to extract data can help avoid random errors; however, caution is still required when extracting data from figures or tables [47-50].

### Assess the Risk of Bias

Assessing the bias of risk involves evaluating the internal validity of studies included in research. For randomized controlled trials, tools such as Risk of Bias (RoB) [62] or its updated version RoB 2 [63] are typically used, with an estimated review time of 10-15 minutes per trial. However, automated tools such as RobotReviewer can streamline the extraction and evaluation process in batches [51-53], thereby improving efficiency, although manual verification is still necessary. Additionally, chatbots based on LLMs can aid in risk of bias assessment (see Multimedia Appendix 8), and their accuracy appears to be comparable to that of human evaluations [23].

### Analyze the Data/Meta-Analysis

Data analysis serves as the source of systematic review results, typically encompassing basic information and outcome findings. The meta-analysis may be one outcome, along with potential components such as subgroup analysis, sensitivity analysis, meta-regression, and detection of publication bias. Numerous software options are available to facilitate these data analyses, including Stata, RevMan, Rstudio, and others [43]. Currently, it appears that chatbots based on LLMs may not fully execute data analysis independently, although they can extract the relevant information. Subsequently, one can employ corresponding software for comprehensive data analysis. Alternatively, after extracting information with chatbots, the ChatGPT Code Interpreter can assist in analysis and generating graphical results, although this requires a subscription to ChatGPT Plus. Moreover, an LLM markedly accelerates the data analysis process, empowering researchers to handle larger data sets with greater efficacy [54].

### Draft the Full Manuscript

The complete drafting of systematic reviews and meta-analyses should adhere to the PRISMA reporting guidelines [64]. It is not advisable to use chatbots such as ChatGPT for article composition. On the one hand, the accuracy and integrity of content generated by ChatGPT require human verification. On the other hand, various research types and journals have different requirements for full-length articles, making it challenging to achieve uniformity in the generated content. However, using tools such as GPT for language refinement and adjusting the content logic can be considered to enhance the quality and readability of the article [33,55]. It is important to declare the use of GPT-related tools in the methods, acknowledgments, or appendices of the article to ensure transparency [24,65].

### Submit and Publish

Submission and publication represent the final steps in the process of conducting systematic reviews and meta-analyses, aside from subsequent updates. At this stage, the potential role of LLM-based tools is to assist authors in recommending suitable journals (see Multimedia Appendix 9). These tools might also aid in crafting components required along with submission of the manuscript such as cover letters and highlights [59]. However, it is imperative to emphasize that the content generated by these tools requires manual verification to ensure accuracy, and all authors should be accountable for the content generated by LLMs.

## Benefits and Drawbacks of Using LLMs

Systematic reviews and meta-analyses are crucial evidence types that support the development of guidelines [3]. The benefits of employing LLM-based chatbots in the production of systematic reviews and meta-analyses include increased speed, such as in the stages of evidence searching, data extraction, and assessment of bias risk; these tools can also enhance accuracy by reducing human errors such as those made while extracting essential information and pooling data. However, there are also drawbacks of these applications of LLMs, such as the potential for generating hallucinations, the requirement for human verification owing to the poor reliability of the models, and that the entire systematic review process is not replicable. Moreover, when interacting with LLM chatbots, it is important to manage data privacy. In particular, when using LLMs to analyze data, especially when including personal patient information, ethical approval and management must be properly addressed.

## Challenges and Solutions

While LLMs can assist in accelerating the production of systematic reviews and meta-analyses in some steps, enhancing accuracy and transparency, and saving resources, they also face several challenges. For instance, LLMs cannot promptly update their versions and information. For example, ChatGPT 3.5 has been trained on data available in 2021. Thus, limitations such as the length of prompts and token constraints, as well as restrictions related to context associations, may potentially impact the overall results and user experience [25]. Although LLM-based autonomous agents have made strides in tasks related to systematic reviews and meta-analyses, their applications are still associated with various issues related to personalization, updating knowledge, strategic planning, and complex problem-solving. The development of LLM-driven autonomous agents adept at systematic reviews and meta-analyses warrants further exploration [66]. The use of LLMs as centrally controlled intelligent agents encompasses the ability to handle precise literature screening, extract and analyze complex data, and assist in manuscript composition, as highlighted by proof-of-concept demonstrations such as MetaGPT [67]. Moreover, the continuous growth of the use of LLMs can pose a significant challenge in ensuring the accuracy of information provided in systematic reviews, particularly if LLMs are indiscriminately overused.

To better facilitate the use of tools such as ChatGPT in systematic reviews and meta-analyses, we believe that, first and foremost, authors should understand the scope and scenarios for applying ChatGPT, clearly defining which steps can benefit from these tools. Second, for researchers, collaboration with computer scientists and AI engineers is crucial to optimize the prompts and develop integrated tools based on LLMs, such as web applications. These tools can assist in seamless transitions between different tasks in the systematic review process. Lastly, for journal editors, collaboration with authors and reviewers is essential to adhere to reporting and ethical principles associated with the use of GPT and similar tools [24,68]. This collaboration aims to promote transparency and integrity, while preventing indiscriminate overuse in the application of LLMs in systematic reviews and meta-analyses.

## Future Perspectives and Conclusion

The emergence of LLMs could have a significant impact on the production of systematic reviews and meta-analyses. In this process, the application of chatbots such as ChatGPT has the potential to speed up certain steps such as literature screening, data extraction, and risk of bias assessment, which are processes that typically consume a considerable amount of time. However, it is important to note that if AI methods such as GPT are employed in performing systematic reviews, disclosure and declaration of the use of these tools are essential. This includes specifying the AI tools used, their roles, and the areas of application within the review process, among other relevant information for full disclosure [24]. In this context, developing a reporting guideline is warranted to guide the application of LLM tools in systematic reviews and meta-analyses. Although the PRISMA 2020 guideline briefly addresses the use of automation technologies, its coverage is limited to steps such as screening, and there is a lack of comprehensive guidance on the broader spectrum of applications [64].

## Appendix

Multimedia Appendix 1 Using ChatGPT 4.0 to assist in generating PubMed search strategies for assessing systematic reviews.

Multimedia Appendix 2 The results obtained after searching the PubMed database based on the search strategy generated by ChatGPT.

Multimedia Appendix 3 Using ChatGPT 4.0 to assist in optimizing the clinical question for conducting a systematic review and meta-analysis.

Multimedia Appendix 4 Using ChatGPT 4 to generate PROSPERO (International Prospective Register of Systematic Reviews) registration information.

Multimedia Appendix 5 Proposal of a systematic review and meta-analysis related to exercises for osteoarthritis generated by Claude 3 based on the provided prompts.

Multimedia Appendix 6 The inclusion and exclusion criteria for a systematic review and meta-analysis on exercise therapy for osteoarthritis based on GPT-4.

Multimedia Appendix 7 Using Claude 3 for data extraction from PDF documents: an example with three randomized controlled trials.

Multimedia Appendix 8 Using Claude 3 for risk of bias assessment: an example with two randomized controlled trials.

Multimedia Appendix 9 Using GPT-4 to assist in selecting target journals for submission of a systematic review and meta-analysis.

## Abbreviations

AI: artificial intelligence
API: application programming interface
LLM: large language model
PICO: Population, Intervention, Comparison, Outcome
PRISMA: Preferred Reporting Items for Systematic reviews and Meta-Analyses
RoB: Risk of Bias
