# Supplementary figures and images for: Potential Roles of Large Language Models in the Production of Systematic Reviews and Meta-Analyses
Source: J Med Internet Res. 2024 Jun 25;26:e56780. doi: 10.2196/56780 (PMC11234072; doi:10.2196/56780)

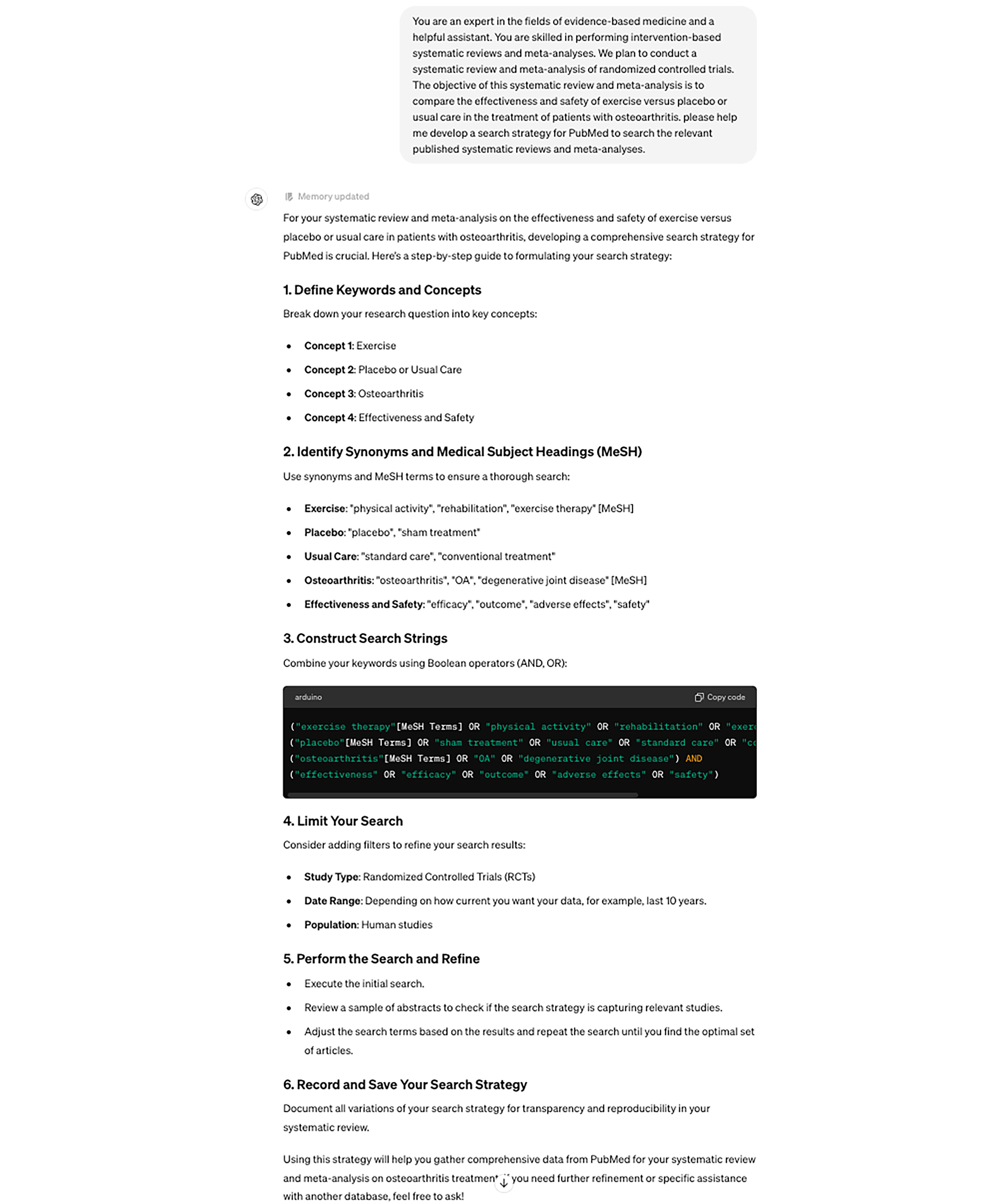

Supplement: Multimedia Appendix 1 [file jmir_v26i1e56780_app1.png]

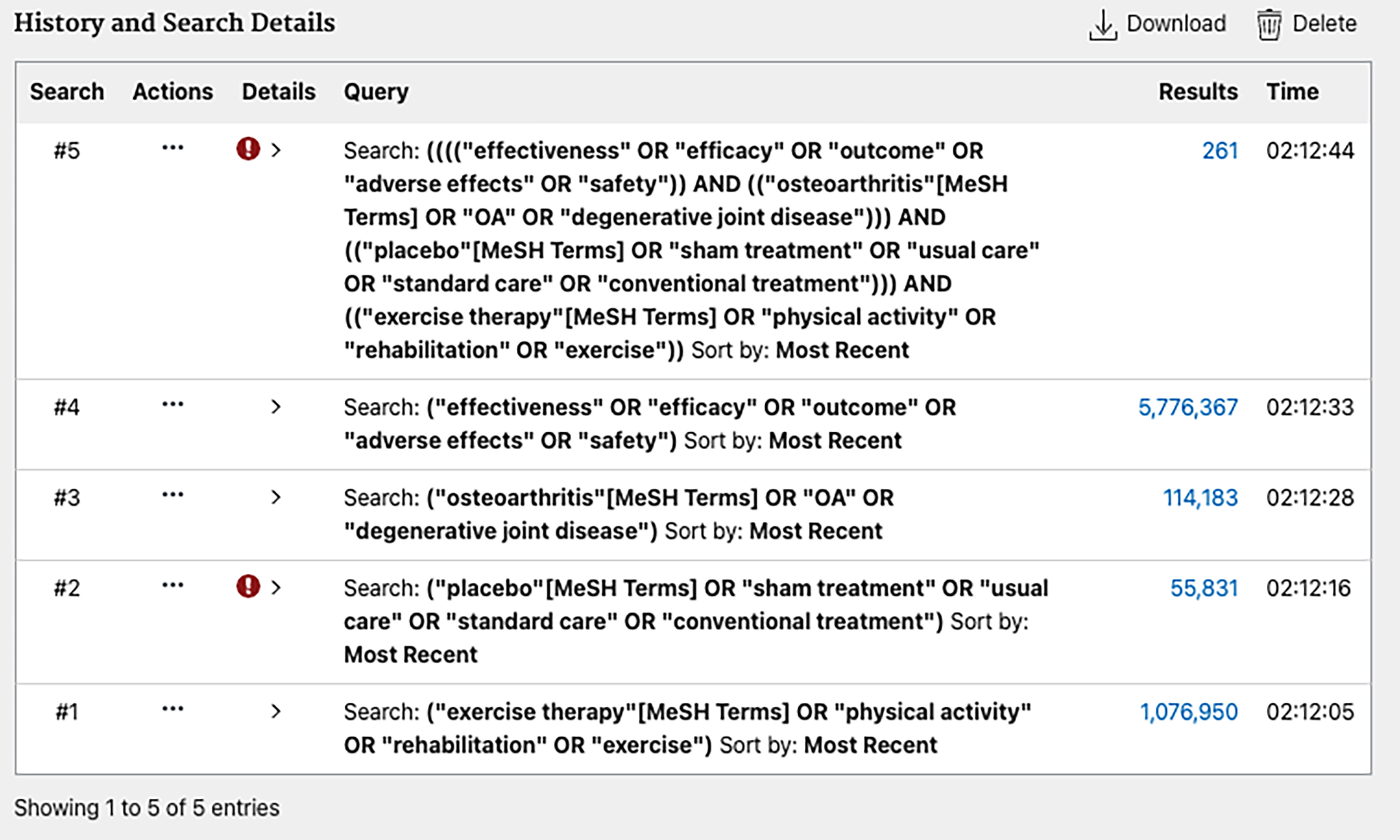

Supplement: Multimedia Appendix 2 [file jmir_v26i1e56780_app2.png]

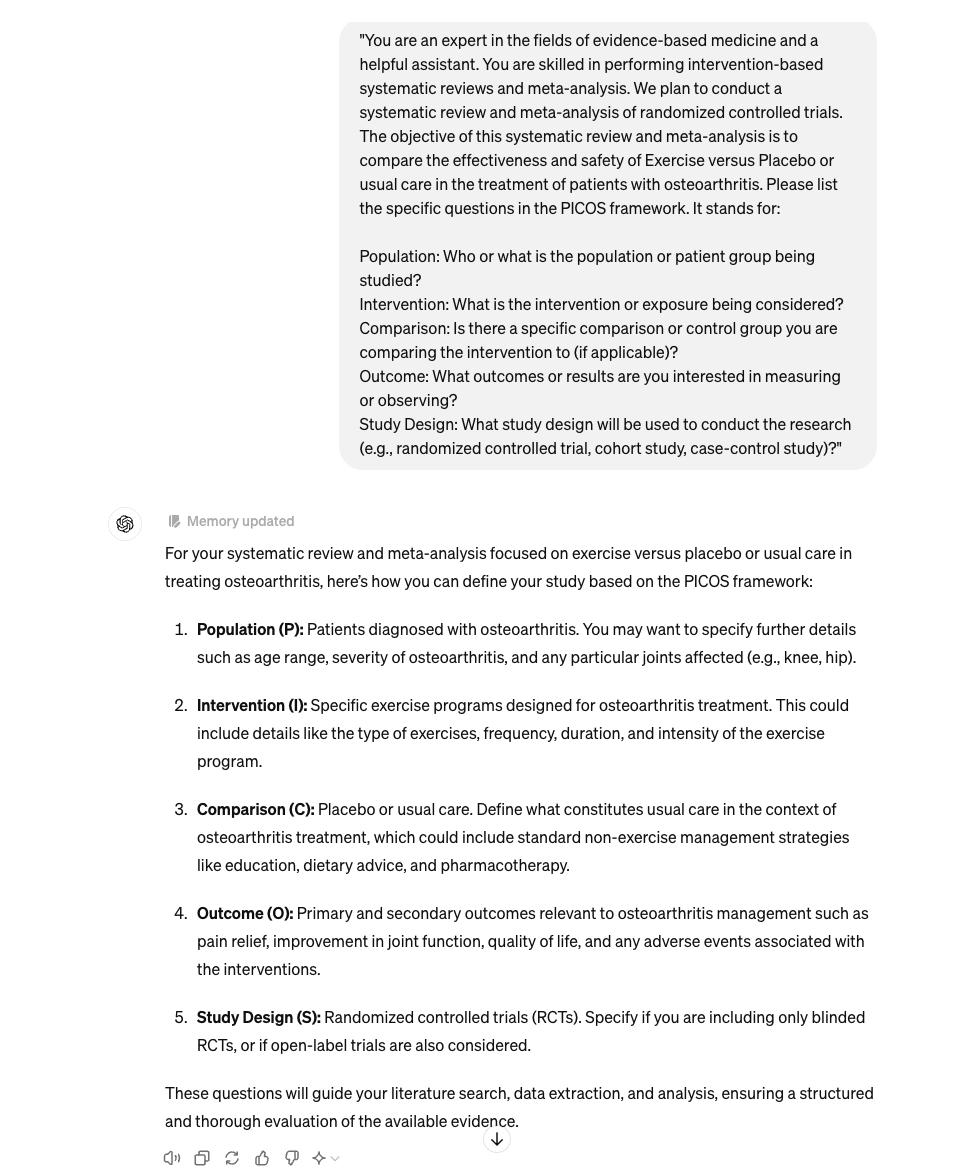

Supplement: Multimedia Appendix 3 [file jmir_v26i1e56780_app3.png]

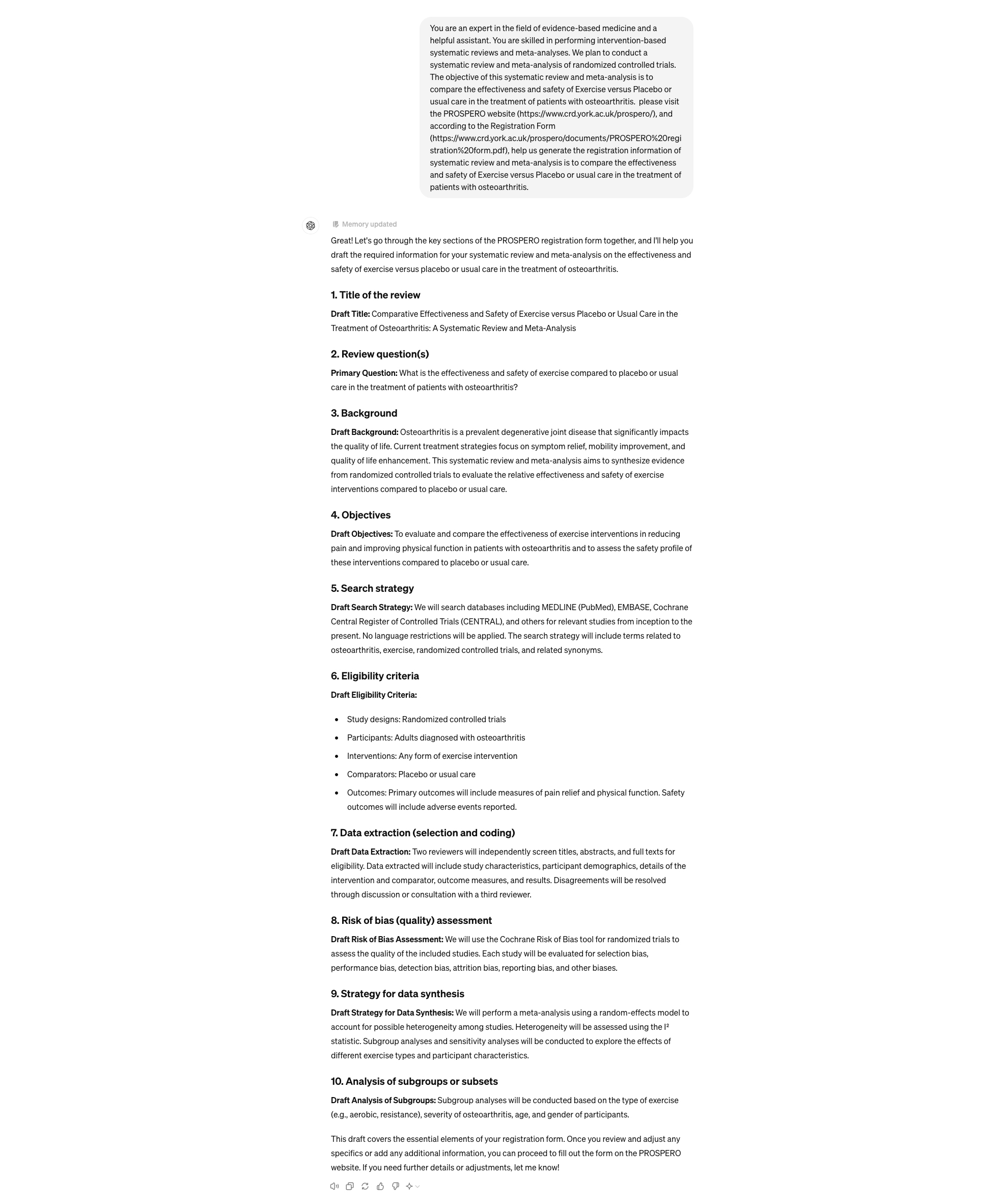

Supplement: Multimedia Appendix 4 [file jmir_v26i1e56780_app4.png]

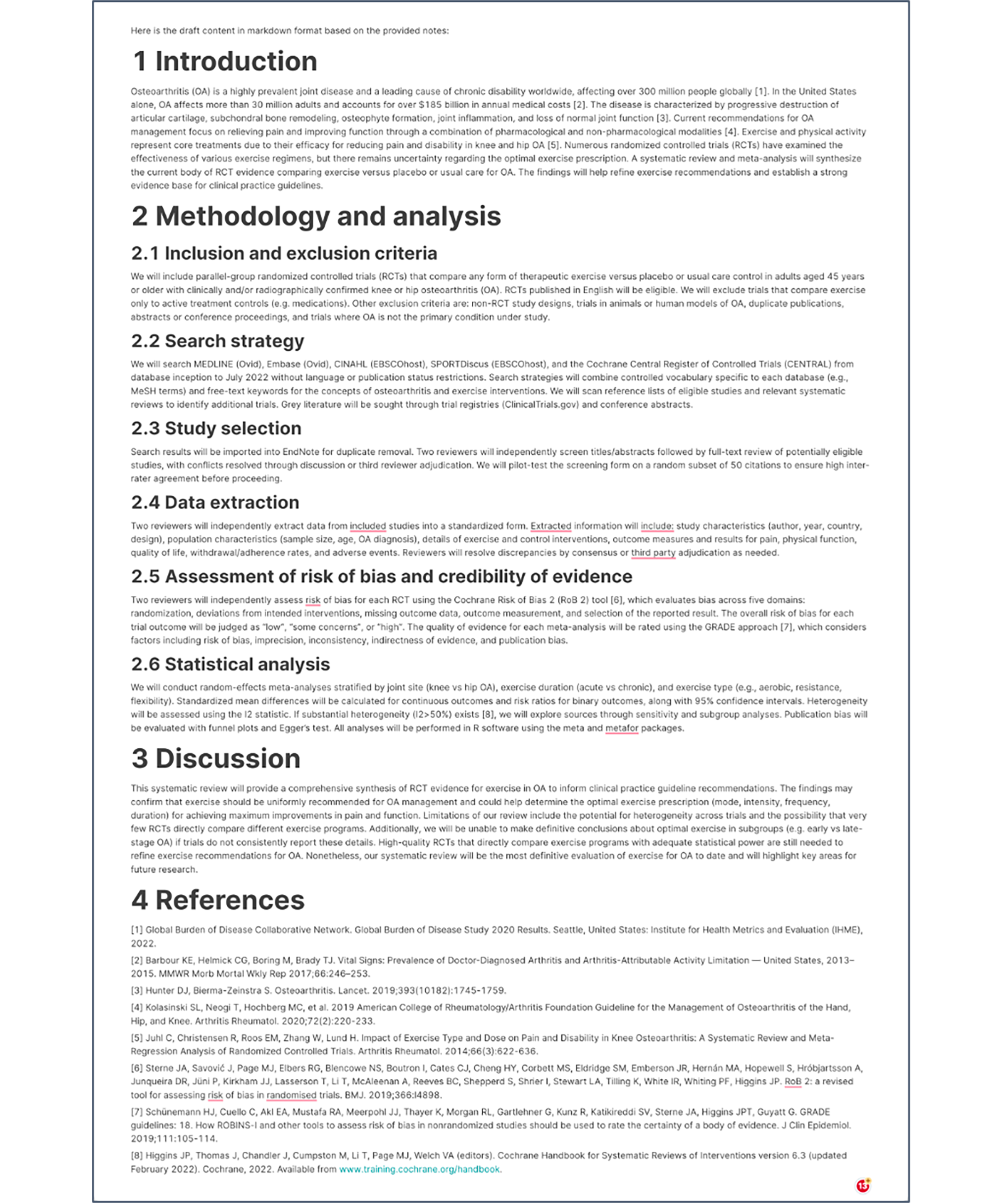

Supplement: Multimedia Appendix 5 [file jmir_v26i1e56780_app5.png]

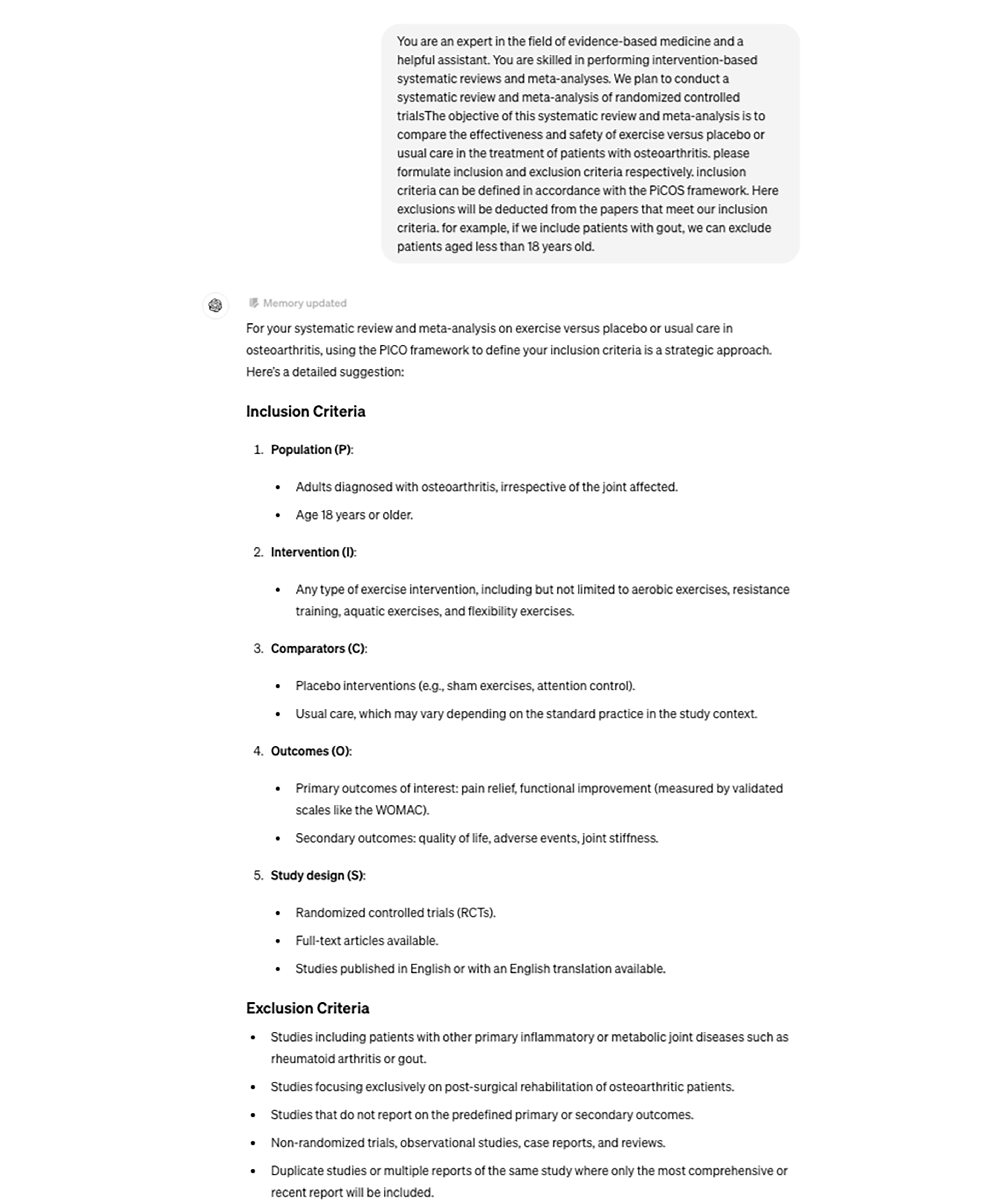

Supplement: Multimedia Appendix 6 [file jmir_v26i1e56780_app6.png]

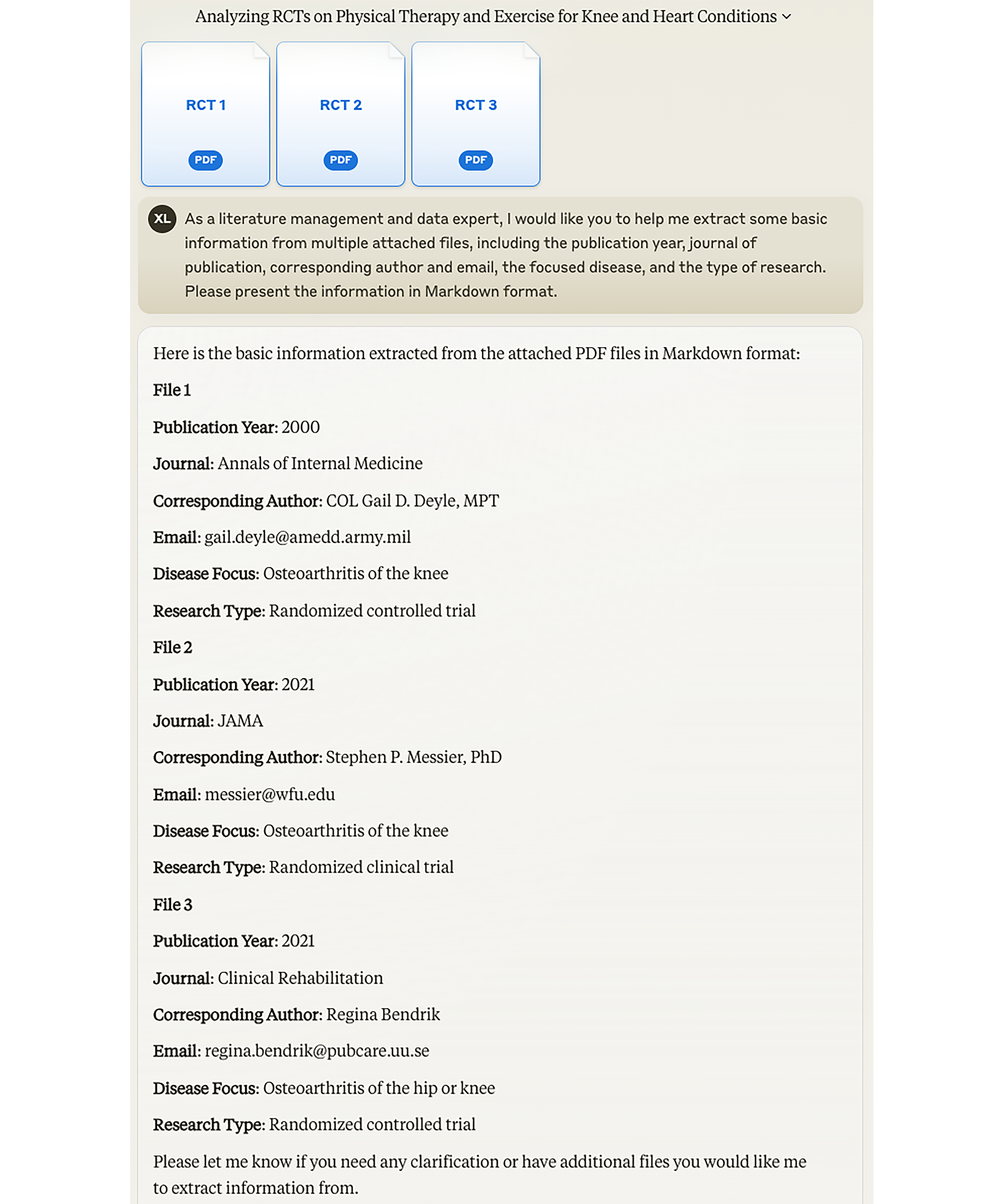

Supplement: Multimedia Appendix 7 [file jmir_v26i1e56780_app7.png]

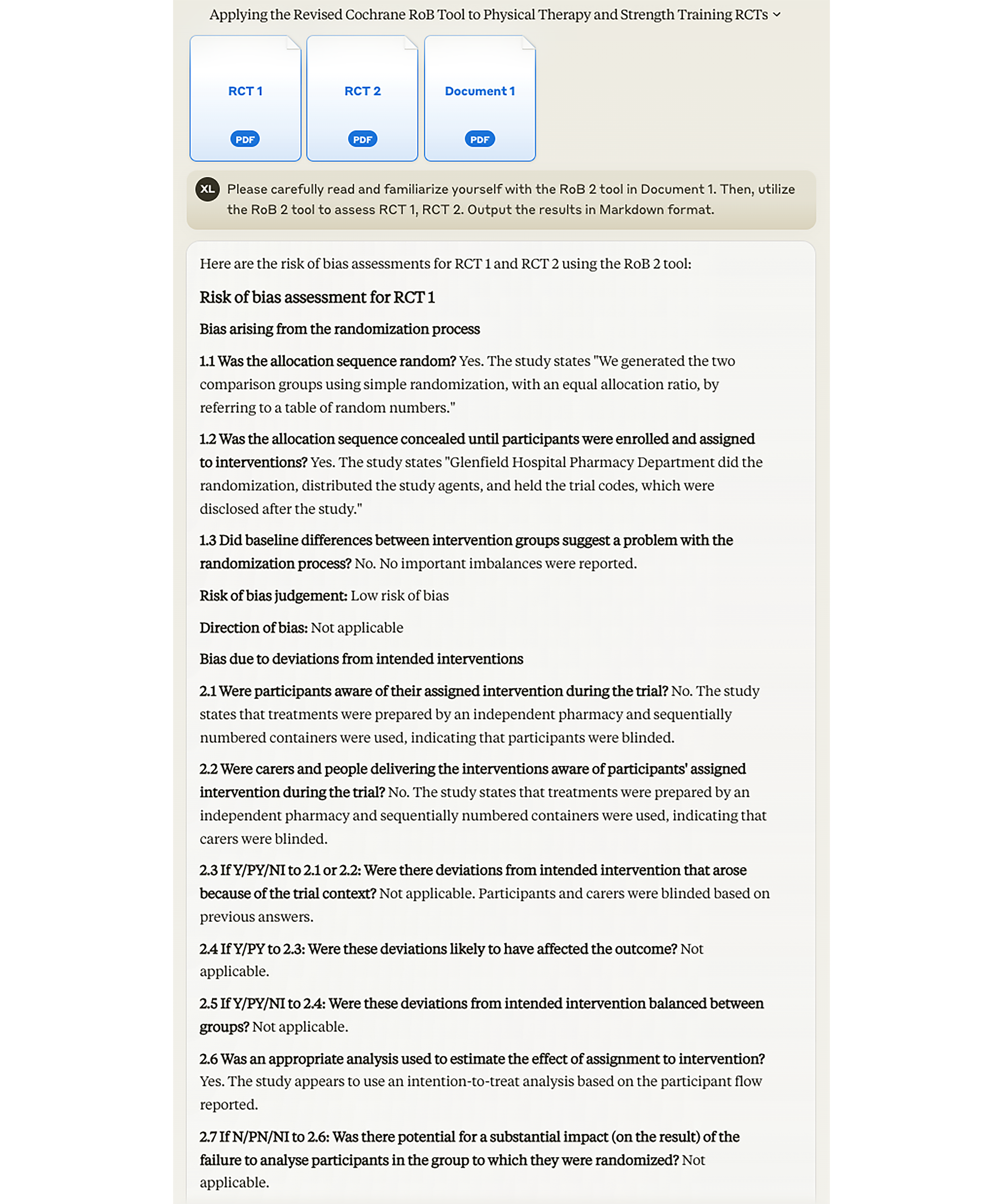

Supplement: Multimedia Appendix 8 [file jmir_v26i1e56780_app8.png]

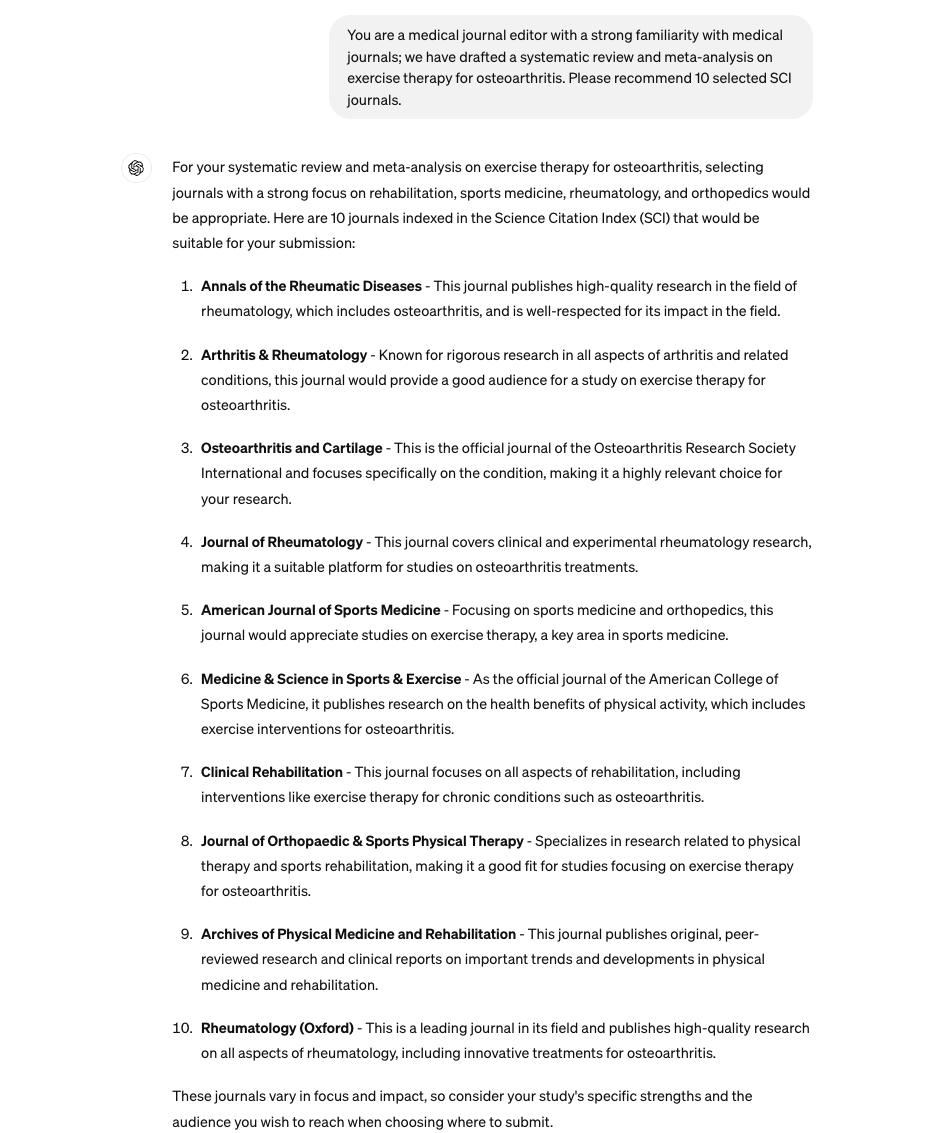

Supplement: Multimedia Appendix 9 [file jmir_v26i1e56780_app9.png]
